# Omalizumab may decrease IgE synthesis by targeting membrane IgE+ human B cells

**DOI:** 10.1186/2045-7022-3-29

**Published:** 2013-09-02

**Authors:** Marcia A Chan, Nicole M Gigliotti, Abby L Dotson, Lanny J Rosenwasser

**Affiliations:** 1 Department of Pediatrics, Division of Immunology Research, Children’s Mercy Hospitals & Clinics, Kansas City, MO 64108, USA; 2 Department of Molecular Biosciences, University of Kansas, Lawrence, KS 66045, USA

**Keywords:** Anti-IgE, IgE, Human B cells

## Abstract

**Background:**

Omalizumab, is a humanized anti-IgE monoclonal antibody used to treat allergic asthma. Decreased serum IgE levels, lower eosinophil and B cell counts have been noted as a result of treatment. In vitro studies and animal models support the hypothesis that omalizumab inhibits IgE synthesis by B cells and causes elimination of IgE-expressing cells either by induction of apoptosis or induction of anergy or tolerance.

**Methods:**

We examined the influence of omalizumab on human tonsillar B cell survival and on the genes involved in IgE synthesis. Tonsillar B cells were stimulated with IL-4 plus anti-CD40 antibody to induce class switch recombination to IgE production in the presence or absence of omalizumab. Cell viability was assessed and RNA extracted to examine specific genes involved in IgE synthesis.

**Conclusions:**

We found that omalizumab reduced viable cell numbers but this was not through induction of apoptosis. IL-4R and germline Cϵ mRNA levels were decreased as well as the number of membrane IgE+ cells in B cells treated with omalizumab. These data suggest that omalizumab may decrease IgE synthesis by human B cells by specifically targeting membrane IgE-bearing B cells and inducing a state of anergy.

## Background

IgE is responsible for type I hypersensitivity reactions such as allergic asthma and allergic rhinitis. Direct correlations have been noted between asthma and serum IgE levels [1]. One approach to controlling allergic diseases has been the development of a monoclonal antibody that binds to IgE. To be effective the anti-IgE must bind only free IgE or membrane IgE present on the surface of B cells. However, the antibody should not bind to IgE bound to either the high affinity or low affinity IgE receptor. One such antibody is omalizumab. Omalizumab (Xolair®; Genentech/Novartis) is a recombinant humanized monoclonal anti-IgE IgG1 kappa antibody used in the treatment of allergic asthma. Omalizumab binds free IgE in serum and membrane-expressed IgE on B cells [2]. In clinical trials, administration of omalizumab either intravenously or subcutaneously significantly reduced serum levels of IgE and asthmatic symptoms [3-5].

Several studies have examined the molecular and cellular targets of omalizumab using samples from allergic asthmatic subjects treated with omalizumab. A significant decrease in IgE secretion and B cell number was noted in cultured peripheral blood mononuclear cells [6]. Also, downregulation of the cytokines IL-2 and IL-13 has been seen in T cells [7]. Djukanovic et al. [8] noted reductions in the numbers of IgE+ stained cells and eosinophils in airway mucosa from bronchial biopsies. Reduced cell numbers of IgE+ stained cells and eosinophils were also seen in nasal biopsy specimens from allergic rhinitis patients treated with omalizumab [9]. The reduction in the number of eosinophils appeared to be due to the induction of apoptosis since the percentage of Annexin V^+^ cells was significantly increased in omalizumab recipients [7]. Although the precise mechanism was not elucidated, it was suggested that elimination of circulating IgE by omalizumab might result in increased apoptosis of eosinophils. An apoptotic mechanism has also been proposed as a mechanism of action by omalizumab to account for the reduction in IgE synthesis by membrane IgE-bearing B cells [10].

In the present study we examined the influence of omalizumab on human tonsillar B cell survival and on the genes involved in IgE regulation. B cells produce IgE after heavy chain class switching from IgM, IgG or IgA and this is mediated by IL-4 or IL-13 [11,12]. Our results show that omalizumab reduced the number of membrane IgE-bearing B cells induced by stimulation using IL-4 plus anti-CD40. However this was not due to the induction of apoptosis but rather may be the result of induction of anergy.

## Methods

### Ethics statement

Tonsillar tissue from 27 tonsils from children under the age of 10 yrs was obtained from the Department of Pathology at Children’s Mercy Hospital, Kansas City, MO within 4 - 6 h of tonsillectomy. The tissues utilized were those that were in excess of clinical needs and would have otherwise been discarded. All identifiers were removed by pathology staff from the tissue before the tissue was received and processed by our laboratory. The protocol was approved by the Pediatric Institutional Review Board of Children’s Mercy Hospitals and Clinics. Since the tissues were deemed to be scavenged specimens the Pediatric Institutional Review Board of Children’s Mercy Hospitals and Clinics waived the need for written informed consent from the participants and classified the protocol to be non-human subjects research.

### Cell isolation

B cells were isolated from tonsillar tissue as we have routinely done and described [13]. Briefly, tonsillar tissue was teased apart and cells diluted in HBSS (Gibco/Invitrogen, Carlsbad, CA). Mononuclear cells were then isolated by Ficoll-Paque™ (GE Healthcare Bioscience AB, Piscataway, NJ) density gradient centrifugation and subsequently B cells were obtained using a B cell enrichment kit (EASY SEP, Stemcell Technologies, Vancouver, BC, Canada). In some instances B cells were further separated into naïve and memory subsets using naïve and memory B cell isolation kits (Stemcell Technologies). All cells were cultured in RPMI1640 supplemented with 10% FBS (Hyclone, Logan UT), 2 mM glutamine (Gibco/Invitrogen), 100 U/mL penicillin (Gibco/Invitrogen), and 100 μg/mL streptomycin (Gibco/Invitrogen). The percentage of CD19+ cells ranged from 98.9 - 99.9% after B cell enrichment.

### Cell stimulation

Tonsillar B cells at a concentration of 1 × 10^6^/mL in supplemented RPMI1640 were treated with 400 U/mL recombinant IL-4 (eBioscience, San Diego, CA) plus 1 μg/mL anti-CD40 (eBioscience) in the presence or absence of omalizumab (Novartis Pharmaceuticals Corp, East Hanover, NJ) or control antibody (human IgGκ) (Southern Biotech, Birmingham, AL). Omalizumab was reconstituted in sterile PBS at a concentration of 15 mg/mL. Dilutions of omalizumab were made in RPMI1640. In some instances the cells were pre-treated with FcR blocking reagent (Miltenyi, Auburn, CA). At the indicated times, viable cell numbers were enumerated by their ability to exclude the dye trypan blue (Sigma–Aldrich, St. Louis, MO). Culture supernatant fluid was harvested for IL-6 quantification by ELISA (eBioscience).

### Quantitative polymerase chain reaction (qRT-PCR)

Tonsillar B cells at a concentration of 1 × 10^6^/mL were left non-treated or were treated as described above. Cytoplasmic RNA was extracted from the cells using the RNeasy Mini kit (Qiagen, Valencia, CA) and cDNA was subsequently synthesized using the High Capacity cDNA kit (Applied Biosystems, Foster City, CA). Quantitative PCR reactions were set up in optical 96-well reaction plates in a final volume of 25 μL containing 12.5 μL of 2 × SYBR® Green master mix (SABiosciences, Frederick, MD), sterile water, 50 ng cDNA and 200 nM IL-4R alpha primers (SABiosciences) or GAPDH primers, (SABiosciences) or 300 nM germline Cϵ primers (forward 5’ – CACATCCACAGGCACCAAAT – 3’; reverse 5’ – ATCACCGGCTCCGGGAAGTA – 3’, IDT, Coralville, IA), or CD23a primers (forward 5’ – CCATGGAGGAAGGTCAATATT – 3’; reverse 5’ – CTCTCTTCCAGCTGTTTTAGA – 3’, IDT) or CD23b primers (forward 5’ – CATAATGAATCCTCCAAGCCA – 3’; reverse 5’ – CTTGGAAACTTGAGAGACGTT – 3’ IDT). PCR cycling conditions were 95°C for 10 min and then 40 cycles of 95°C for 15 sec, and 60°C for 1 min using a 7500 Real Time PCR System (Applied Biosystems). For each sample the gene of interest was normalized to GAPDH using the ∆∆C_t_ method to calculate relative fold difference in mRNA levels compared to non-treated samples. Comparisons between groups were made using the paired t-test, p < 0.05.

### Flow cytometry

Purity of B cells, cell viability and apoptotic cells were evaluated by flow cytometry (Accuri Cytometers Inc., Ann Arbor, MI) using anti-human CD19 conjugated to PE, (clone HIB19), APC-conjugated anti-human CD27 (clone M-T271), FITC-conjugated anti-human IgD (clone IAb-2), 7-AAD and PE-conjugated Annexin V (BD Pharmingen, San Diego, CA), respectively. The cells were stained using 15 μL of each antibody per million cells. To evaluate proliferation the cells were stained with 2.5 μM CFSE (Invitrogen) prior to stimulation with IL-4 plus anti-CD40 in the presence or absence of omalizumab. For surface IgE expression cells were stained with either 15 μL/10^6^ cells PE-conjugated anti-human IgE (clone MHE-18, BioLegend, San Diego, CA) or 10 μL/10^6^ cells APC-conjugated anti-human IgE (clone MB10-5C4, Miltenyi Biotech, Auburn, CA) as indicated. Flow cytometric analysis was performed using Accuri C6-CFlow software (Accuri Cytometer Inc.). Unstained cells or non-treated cells (Annexin V/7-AAD, CFSE experiments) were used to establish gates.

## Results and discussion

### Omalizumab reduced the number of membrane IgE+ B cells

**Figure 1:**
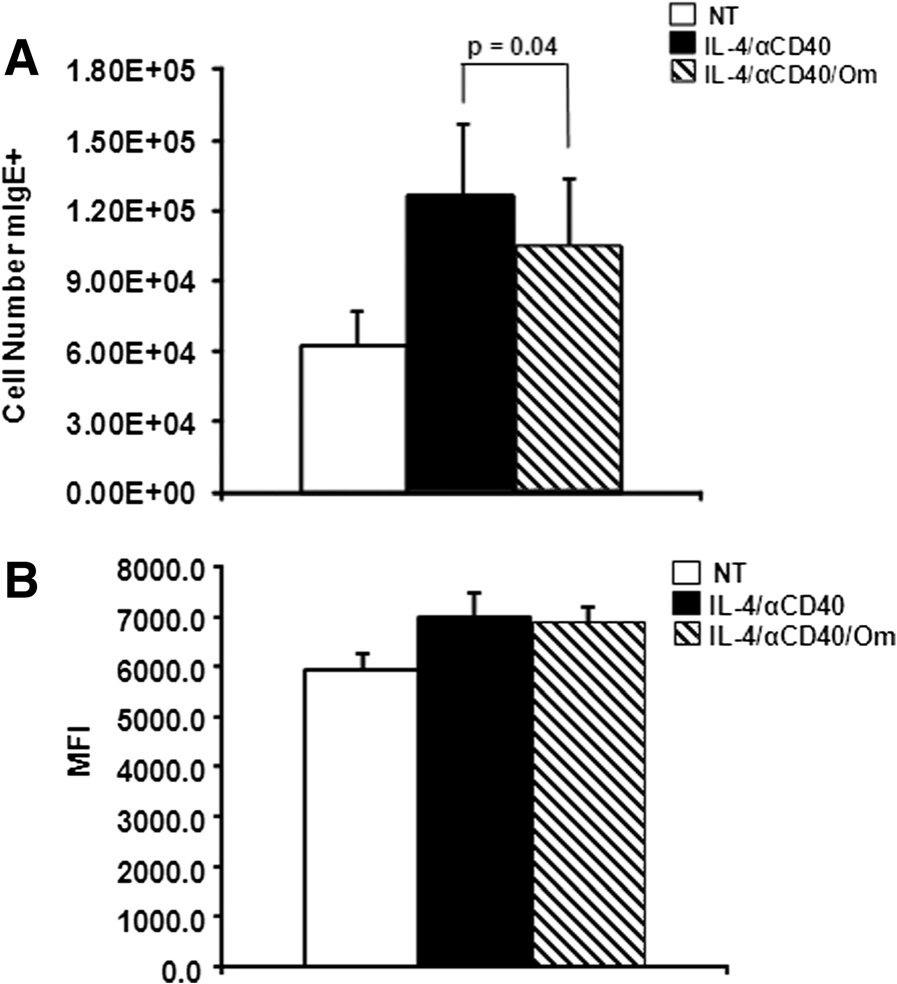
**Omalizumab reduced the number of membrane IgE+ B cells.** B cells were left non-treated (NT) or treated with IL-4 + anti-CD40 in the presence or absence of 1 μg/mL omalizumab (Om). After 4 days B cells were stained with PE-conjugated anti-human IgE. **(A)** Number of mIgE+ cells. **(B)** Mean fluorescence intensity. Data are expressed as the mean ± SEM (n = 6 tonsils). Statistically significant difference (p value) was determined by t-test.

The combination of IL-4 plus anti-CD40 is known to induce class switching to secretion of IgE by human B cells [11,12]. To determine if omalizumab influenced the ability of IL-4 plus anti-CD40 to induce B cells to synthesize IgE, B cells were evaluated by flow cytometry for the number of membrane IgE+ cells after treatment for 4 days with IL-4 plus anti-CD40 in the presence or absence of omalizumab. Figure 1A illustrates that omalizumab significantly decreased the number of membrane IgE+ cells (p = 0.04) by 17%. The strength of membrane IgE expression in each cell group was compared by noting the mean fluorescence intensity (MFI) of the detecting anti-IgE antibody. No differences among the three cell groups were observed (Figure 1B). As a control, Figure 1B suggested that the decrease in number (Figure 1A) of membrane IgE+ cells was not likely due to competition between omalizumab and PE-conjugated anti-human IgE for membrane IgE binding sites. The results of Figure 1 suggest that omalizumab had a direct effect on the B cells and could reduce the number of membrane IgE+ cells present in IL-4 plus anti-CD40 treated B cell cultures.

### Omalizumab reduced IL-6 secretion in human B cells

**Figure 2:**
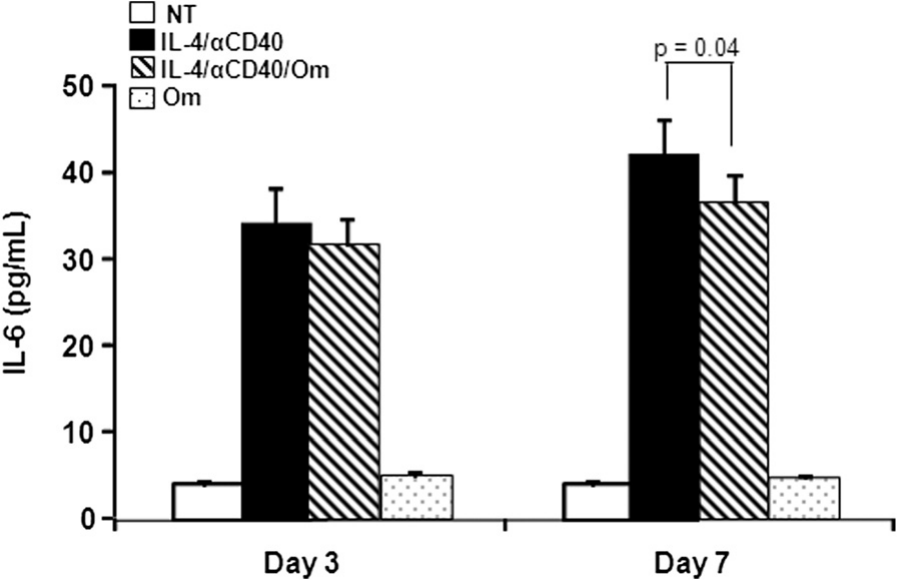
**Omallizumab reduced IL-6 secretion in human B cells.** B cells were left non-treated (NT), treated with IL-4 + anti-CD40 in the presence or absence of 1 μg/mL omalizumab (Om) or omalizumab alone. After 3 and 7 days, culture supernatant fluid was collected and quantified for IL-6 by ELISA. Data are expressed as the mean ± SEM (n = 6 tonsils). Statistically significant difference (p value) was determined by t-test.

Treatment of human B cells with IL-4 plus anti-CD40 leads to IL-6 secretion within 48 h [14]. It recently was reported that omalizumab reduced IL-6 and IL-8 secretion in airway smooth muscle cells [15] and in human skin mast cells [16]. We sought to determine if omalizumab could interfere with the IL-6 production induced by IL-4 plus anti-CD40 in B cells. Figure 2 shows that IL-6 production occurred in B cells after 3 and 7 days of stimulation with IL-4 plus anti-CD40. The IL-6 level was unaffected at day 3 by omalizumab but by day 7, the level had been reduced by ~15%. Omalizumab reduced the amount secreted from 42.65 pg/mL to 36.50 pg/mL (p = 0.04 at day 7). Thus, omalizumab can affect the IL-6 secretion induced by IL-4 plus anti-CD40. The reduced cell number (Figure 1) of 17% was similar to the amount of IL-6 lost in Figure 2. One interpretation would be that the decreased IL-6 was due to a loss of IL-6-producing cells rather than a general down regulation of IL-6.

### Omalizumab decreased B cell numbers

**Figure 3:**
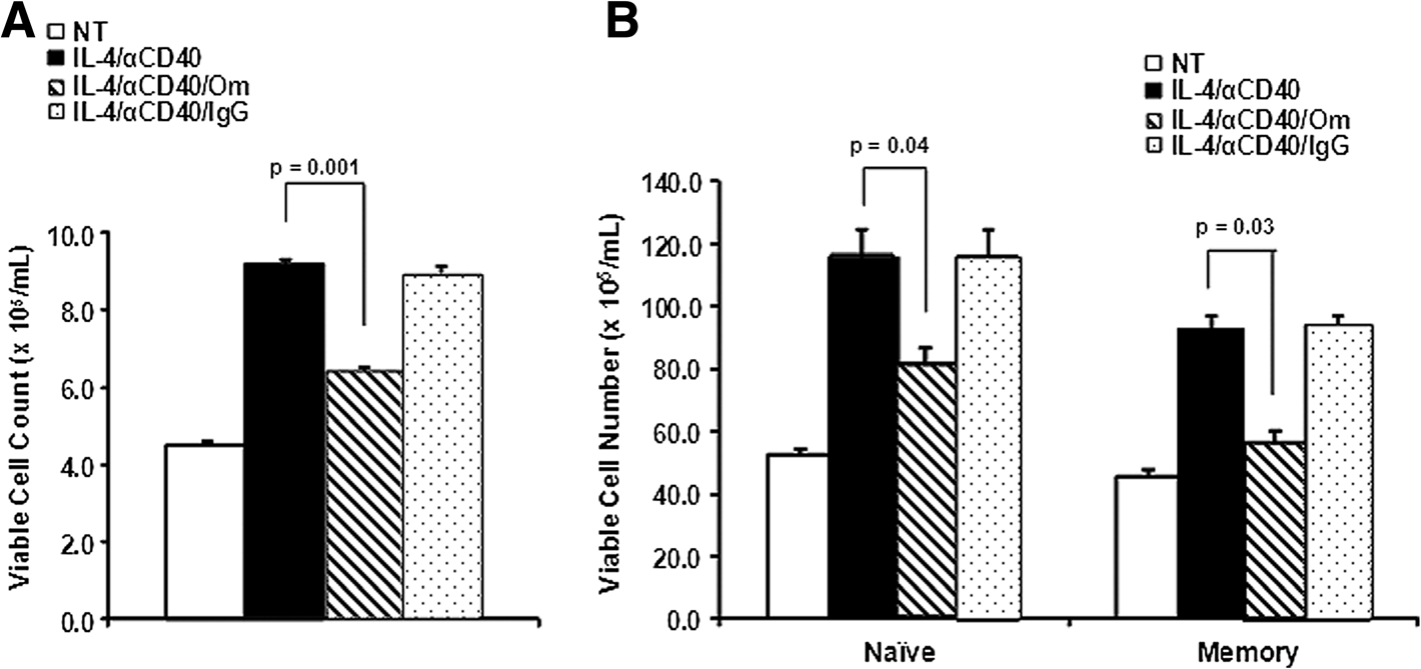
**Omalizumab reduced viable human B cell numbers.** Total B cells **(A)** and naïve and memory B cell subsets **(B)** were left non-treated (NT) or treated with IL-4 + anti-CD40 in the presence or absence of 1 μg/mL omalizumab (Om) or human IgG. Live cells were enumerated by trypan blue exclusion 3 days later. Data are expressed as the mean ± SEM (n = 7 tonsils, panel **A**; n = 4 tonsils, panel **B**). Statistically significant differences (p values) were determined by t-test.

Since we observed a reduction in the number of membrane IgE+ cells in the presence of omalizumab and it was suggested previously that omalizumab may interact with IgE-bearing cells leading to lysis of these cells [10], we sought to determine if omalizumab could affect the viability of IL-4 plus anti-CD40 treated B cells. In our initial experiments we pre-treated the cells with FcR blocking reagent (to block Fc receptor-mediated antibody binding) before treatment with omalizumab since it was possible that omalizumab might bind Fc gamma receptors present on B cells. However, we did not observe any difference in the effect of omalizumab on B cells with or without pretreatment with FcR block (data not shown). Four concentrations (0.1, 0.5, 1.0 and 2.0 μg/mL) of omalizumab were initially tested. Viable cell numbers in response to IL-4 plus anti-CD40 were not significantly different among the concentrations of omalizumab used (data not shown). Thus, we chose to test a single concentration of omalizumab (1 μg/mL) since this concentration also was shown to consistently decrease IgE-induced IL-6 and IL-8 secretion in airway smooth muscle cells [15]. After 3 days of culture, cells were enumerated for viability by trypan blue exclusion. Figure 3A shows that there was a significant decrease in the number of viable B cells cultured with IL-4 plus anti-CD40 in the presence of omalizumab compared to cells cultured with IL-4 plus anti-CD40 alone (p = 0.001). When B cells were cultured with IL-4 plus anti-CD40 in the presence of a control antibody (human IgG1κ) no decrease in viable cell number was seen. Non-treated cells remained viable during the 3 days of culture, and in the presence of omalizumab alone the viable cell number did not change (data not shown). We also examined the effect of omalizumab on subsets of B cells. Naïve and memory B cells were isolated based on expression of IgD and CD27. Figure 3B shows that both naïve and memory B cells were equally sensitive to omalizumab. Significant decreases in viable cells (p =0.04 and p = 0.03, respectively) were observed in both subsets in the presence of omalizumab.

**Figure 4:**
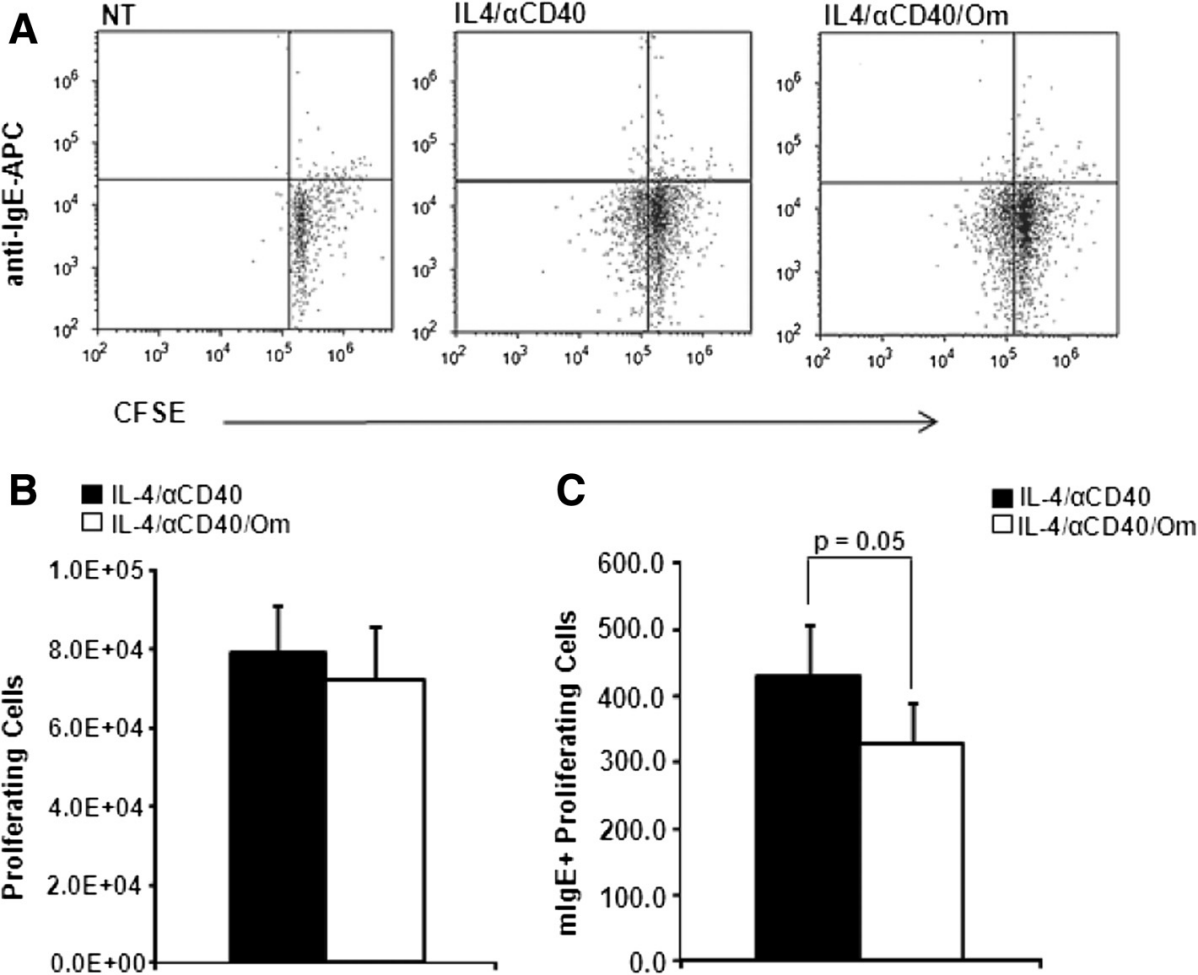
**Omalizumab reduced proliferating mIgE+ B cells.** B cells were labeled with 2.5 μM CFSE prior to treatment with IL-4 + anti-CD40 in the presence or absence of 1 μg/mL omalizumab (Om). After 3 days the cells were stained with APC conjugated anti-human IgE and analyzed by flow cytometry **(A)** for the number of proliferating cells **(B)** and the number of proliferating mIgE+ cells **(C)**. Panel **A**: Representative experiment of 4 independent experiments. NT: non-treated cells. Panels **B** and **C**: Data are expressed as the mean ± SEM from 4 separate independent experiments (4 tonsils). Statistically significant difference (p value) was determined by t-test.

To determine if omalizumab was interfering with cell proliferation, cells were labeled with CFSE before stimulation and the number of proliferating cells analyzed by flow cytometry (Figure 4A). No significant difference in the number of proliferating cells was observed when cells were stimulated for 3 days in the presence of omalizumab (Figure 4B). However, when the small minority of proliferating membrane IgE+ cells was evaluated a significant decrease (p = 0.05) in this subset of cells was observed in the presence of omalizumab (Figure 4C). On average the number of mIgE+ cells was reduced by 25%. These results suggest that the decrease in viable cell number in cultures treated with IL-4 plus anti-CD40 in the presence of omalizumab was most likely due to a form of cell death. In addition, omalizumab appeared to be able to selectively reduce the number of proliferating membrane IgE+ cells in response to IL-4 plus anti-CD40 stimulation.

### Omalizumab did not induce apoptosis in human B cells

**Figure 5:**
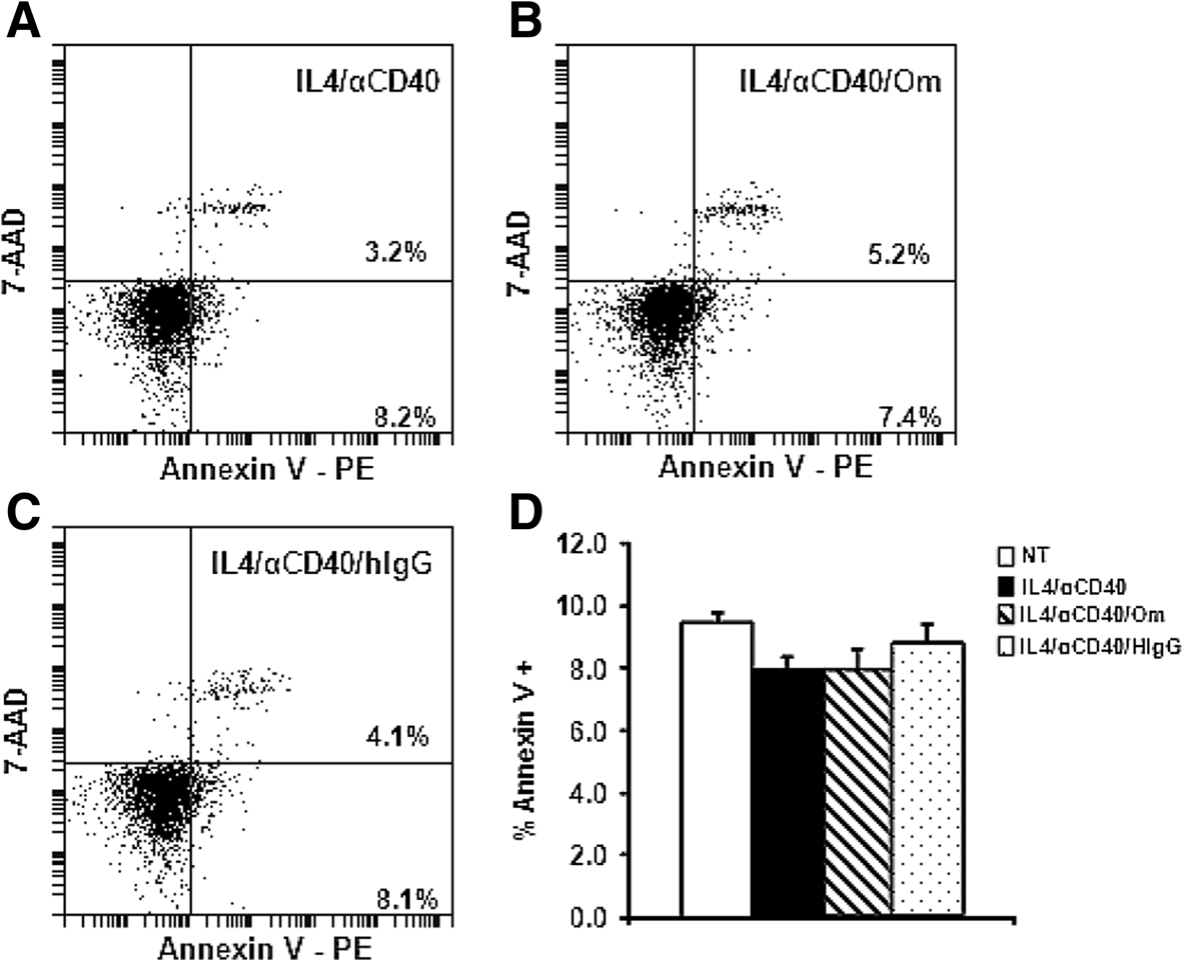
**Omalizumab did not induce apoptosis in human B cells.** B cells were left non-treated (NT) or treated with IL-4 + anti-CD40 in the presence or absence of 1 μg/mL omalizumab (Om) or human IgG. After 3 days B cells were stained with 7-AAD and PE-conjugated Annexin V and analyzed by flow cytometry. Panels **A** – **C**: Representative experiment of 4 independent experiments. Percentage of Annexin V positive cells is indicated in the lower right quadrant. **(A)** IL-4 + anti-CD40 **(B)** IL-4 + anti-CD40 + Om **(C)** IL-4 + anti-CD40 + IgG. Panel **D**: Mean percentage of Annexin V+ cells ± SEM from 4 separate independent experiments (4 tonsils).

We hypothesized that the decrease in cell number observed in cells treated with IL-4 plus anti-CD40 in the presence of omalizumab could be due to the induction of apoptosis. To determine if omalizumab was inducing apoptosis in these cultures, B cells were left non-treated or treated with IL-4 plus anti-CD40 in the presence or absence of omalizumab or control antibody. After 3 days of culture the cells were stained with 7-AAD and PE-conjugated Annexin V and analyzed by flow cytometry. Annexin V is an indicator of apoptotic cells and 7-AAD is excluded by viable cells. Flow cytometric analysis showed that omalizumab did not induce additional Annexin V positivity in human B cells (Figure 5). In a representative experiment, the percentage of Annexin V positive cells was similar in cells treated with IL-4 plus anti-CD40 alone (8.2%) (Figure 5A) and in the presence of omalizumab (7.4%) (Figure 5B) or control antibody (8.1%) (Figure 5C). When four separate independent experiments were analyzed together, the percentage of Annexin V positive cells was similar in all three treatment groups (Figure 5D). These results suggest that the decrease in cell number in B cell cultures treated with IL-4 plus anti-CD40 in the presence of omalizumab was not due to induction of apoptosis.

### Omalizumab reduced IL-4Rα and germline Cϵ mRNA levels in human B cells

**Figure 6:**
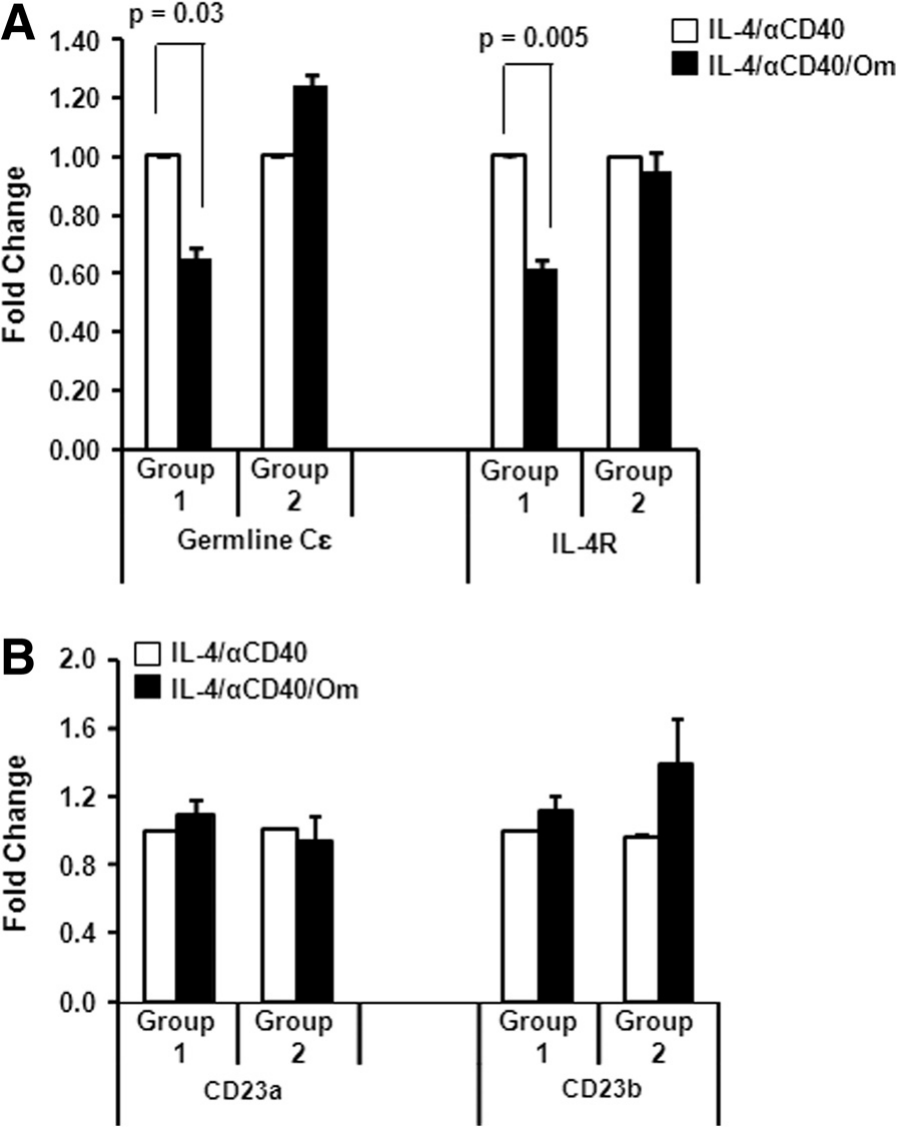
**Omalizumab reduced germline Cϵ and IL-4Rα mRNA levels in ~ 50% of tonsillar samples.** B cells were treated with IL-4 + anti-CD40 in the presence or absence of 1 μg/mL omalizumab (Om). After 3 days RNA was isolated and quantified by qRT-PCR for **(A)** germline Cϵ and IL-4Rα mRNA. **(B)** CD23a and CD23b mRNA. Data are expressed as the mean ± SEM (n = 5 tonsils for group 1 and n = 5 tonsils for group 2). Statistically significant differences (p values) were determined by t-test.

Since omalizumab appeared to reduce the number of membrane IgE+ cells present in cultures treated with IL-4 plus anti-CD40, we sought to determine if omalizumab could affect the potential of B cells to commit to IgE synthesis. To do this we examined the mRNA levels of IL-4Rα and germline Cϵ since IL-4 is required for switch recombination and germline Cϵ precedes final recombination switching to the mature Cϵ transcript. B cells were left non-treated or treated with IL-4 plus anti-CD40 in the presence or absence of omalizumab. Cytoplasmic RNA was isolated from these cells 72 h later and cDNA prepared. Quantitative RT-PCR was used to evaluate mRNA levels using specific primers for IL-4Rα chain and germline Cϵ. The IL-4Rα chain is a shared subunit of both type I and type II IL-4 receptors expressed on B cells [17,18]. The data identified two groups of samples according to responsiveness to omalizumab. Figure 6A shows that omalizumab significantly reduced both IL-4Rα chain and germline Cϵ mRNA levels in 50% of cultures treated with IL-4 plus anti-CD40. In Group 1 (n = 5) omalizumab significantly reduced germline Cϵ (p = 0.03) and IL-4Rα (p = 0.005) mRNA levels by 36% and 39%, respectively. In Group 2 (n = 5) there was a slight enhancement (though not statistically significant) of germline Cϵ mRNA and no change in IL-4Rα mRNA levels in the presence of omalizumab.

The low affinity receptor for IgE (FcϵR2, CD23) is known to play an important role in IgE regulation [19]. CD23 exists in soluble and membrane forms. The soluble form of CD23 appears to positively regulate IgE synthesis by B cells whereas the membrane form participates in a negative-feedback mechanism [20]. Human B cells express both isoforms of CD23 (CD23a and CD23b). We as well as others have shown that IL-4 can up-regulate the expression of CD23 on human B cells [21,22]. Since we observed changes in IL-4R expression in the presence of omalizumab we examined the effect of omalizumab on CD23 mRNA expression. Omalizumab did not affect the up-regulation of either isoform of CD23 on human B cells mediated by IL-4 (Figure 6B). No statistically significant difference in either CD23a or CD23b mRNA levels was observed in either group treated with IL-4 plus anti-CD40 in the presence of omalizumab. Taken together these results suggest that omalizumab reduced the levels of IL-4Rα chain and germline Cϵ mRNA in cultures treated with IL-4 plus anti-CD40 but reduction was seen in only 50% of the tonsillar samples tested. In contrast, levels of CD23 mRNA were unaffected by the presence of omalizumab in both groups of tonsillar samples.

## Conclusions

Omalizumab is a recombinant DNA-derived humanized anti-IgE antibody and has been used successfully to treat patients with moderate-to-severe, and severe persistent allergic asthma [3,4,23]. Also, patients with allergic rhinitis showed marked improvement of symptoms when treated with omalizumab [24].

Omalizumab binds circulating IgE, and membrane IgE on B cells, and prevents subsequent IgE-driven responses such as the release of inflammatory mediators histamine and leukotrienes by basophils and mast cells. However, omalizumab does not bind IgE when IgE is bound to either FcϵRI or CD23 [2]. In addition, omalizumab appears to display several other anti-inflammatory properties [2,6,8]. In allergic asthmatics treated with omalizumab a significant decrease in cellular sensitivity to allergen challenge was observed [25] as well as decreases in circulating serum levels of IL-5, IL-8 and IL-13 [26]. Peripheral blood and sputum eosinophil counts [8,26] were decreased in omalizumab- treated asthmatics and it was proposed that the reduction in eosinophil counts was due to the induction of apoptosis in these cells [7]. Also, omalizumab significantly reduced RNA and protein levels of IL-6, IL-8,TNFα and IL-4 in a dose-dependent manner in IgE-stimulated human airway smooth muscle cells isolated from biospsy specimens of patients with asthma, chronic obstructive pulmonary disease and healthy controls [15]. Finally, a significant reduction in the number of B cells [6] and the number of mIgE+ B cells [8] has been observed in patients with allergic asthma after treatment with omalizumab.

It was proposed that omalizumab bound to mIgE on B cells might mimic the pathway that leads to apoptosis or anergy in B cells treated with anti-IgM or anti-IgG [27,28]. In the present work, we have shown that omalizumab appeared to reduce the number of B cells that synthesize IgE. One measure of IgE-producing B cells is expression of IgE containing the membrane associated exons and displayed as an integral membrane surface protein to create membrane IgE+ (mIgE+) B cells. We observed 17% fewer mIgE+ B cells in cultures treated with IL-4 plus anti-CD40 in the presence of omalizumab compared to cultures treated with IL-4 plus anti-CD40 alone. Also in the presence of omalizumab there were fewer proliferating mIgE+ cells. This is similar to the work of others [8] using histochemical analysis of bronchial biopsies from patients with mild to moderate persistent asthma that were treated with omalizumab. A significant reduction in IgE+ cells was noted in samples from these patients compared to the placebo group; however, in that study the IgE+ cells were not further characterized. In our study, five possibilities exist to explain the observed omalizumab-induced decrease in the number of membrane IgE+ B cells: 1) reduced proliferation in response to IL-4 plus anti-CD40; 2) induction of anergy; 3) induction of apoptosis, 4) prevention of existing B cells to class switch to expression of IgE; or 5) omalizumab-induced shedding or internalization of membrane IgE.

We demonstrated that omalizumab reduced cell numbers in B cell cultures treated for 3 days with IL-4 plus anti-CD40. This was shown by enumerating viable cell number by exclusion of the viable dye trypan blue. The loss of cell numbers did not appear to be due to the induction of apoptosis since the percentage of Annexin V positive cells was similar in non-treated cells and cells treated with IL-4 plus anti-CD40 in the presence or absence of omalizumab or control antibody. Thus, in our B cell cultures omalizumab did not appear to induce apoptosis. Rather the reduction in viable cell number may have been the result of the cells entering an anergic state. Anergic B cells have a limited life span of only 3 – 4 days [29].

When mRNA levels of genes involved in IgE synthesis were examined we observed a significant reduction by cultures containing omalizumab of IL-4Rα and germline Cϵ mRNA levels suggesting a downregulation in IL-4 responsiveness. The reduced levels of IL-4Rα and Cϵ mRNA appeared to be selectively targeted by omalizumab since mRNA levels of both isoforms of the low affinity IgE receptor CD23 were unaffected. Roth and Tamm [15] also noted that omalizumab did not affect expression of either high or low affinity IgE receptors by human airway smooth muscle cells cultured *in vitro* with omalizumab. Since we also observed that omalizumab reduced the numbers of mIgE+ B cells in cultures stimulated by IL-4 plus anti-CD40, it is likely that the reduction of IL-4Rα and Cϵ mRNA levels is a direct reflection of the reduced numbers of mIgE+ cells in cultures containing omalizumab.

It was of interest that omalizumab reduced IL-4Rα and germline Cϵ mRNA levels in only 50% of the 10 tonsils analyzed for the gene expression studies. These results suggest that not all individuals may be responsive to omalizumab. Unfortunately, all identifiers were removed from the tonsil tissue before processing so we were unable to determine the allergic status of the individuals from which B cells were responsive to omalizumab. However, our results are in agreement with response rates to omalizumab from clinical trial data. The data indicated that not all patients with persistent asthma who were treated with omalizumab showed improvement [30,31]. The patients were grouped as responders and non-responders and the response rates to omalizumab ranged from 53.1% to 68.5%.

## Competing interests

The authors declare that they have no competing interests.

## Authors’ contributions

MC contributed to the conception and design of experiments, data acquisition and analysis, interpretation of data and drafted the manuscript. NG carried out the flow cytometry experiments and analysis and the molecular studies. AD carried out the immunoassays. LR participated in the design of the study and contributed to the interpretation of data. All authors read and approved the final manuscript.
